# Potential of circulating tumor DNA as a predictor of therapeutic responses to immune checkpoint blockades in metastatic renal cell carcinoma

**DOI:** 10.1038/s41598-021-85099-4

**Published:** 2021-03-10

**Authors:** Yeon Jeong Kim, Yumi Kang, Jun Seop Kim, Hyun Hwan Sung, Hwang Gyun Jeon, Byong Chang Jeong, Seong Il Seo, Seong Soo Jeon, Hyun Moo Lee, Donghyun Park, Woong-Yang Park, Minyong Kang

**Affiliations:** 1 Samsung Genome Institute, Samsung Medical Center, Seoul, South Korea; 2 Department of Urology, Samsung Medical Center, Sungkyunkwan University School of Medicine, 81 Irwon-ro, Gangnam-gu, Seoul, 06351 South Korea; 3 Geninus, Seoul, South Korea; 4 Department of Molecular Cell Biology, Sungkyunkwan University School of Medicine, Suwon, South Korea; 5 Department of Health Sciences and Technology, Seoul, South Korea; 6 Department of Digital Health, SAIHST, Sungkyunkwan University, Seoul, South Korea

**Keywords:** Biomarkers, Molecular medicine, Oncology, Urology

## Abstract

We evaluated the predictive role of circulating tumor DNA (ctDNA) detection by targeted deep sequencing in patients with metastatic renal cell carcinoma (mRCC) treated with immune checkpoint blockades (ICB). To determine the feasibility of ctDNA detection in our panel encompassing 40 genes, we collected 10 ml of blood from 20 patients at the time of radical nephrectomy. We analyzed somatic mutations in primary tumors and ctDNA samples from these patients. We finally collected 10 ml of blood before and after 1 month of treatment, respectively, from four patients with mRCC who received first-line ICB treatment. Variants were detected in primary tumors of 15 patients (75%) and ctDNA was detected in the plasma of 9 patients (45%). We examined the predictive role of ctDNA in four patients who received first-line ICB therapy. In two patients showing partial response, ctDNA levels decreased after 1 month of ICB treatment. However, in one patient who showed disease progression, ctDNA levels increased after 1 month of ICB treatment. Taken together, ctDNA detection in plasma by targeted deep sequencing was feasible in patients with RCC. Moreover, the levels of ctDNA could be an early predictor of treatment response in patients with mRCC who receive ICB therapy.

## Introduction

Over the last decade, various promising agents, such as multi-kinase inhibitors and immune checkpoint blockades (ICB), have been approved for treating metastatic renal cell carcinoma (mRCC)^1^. These drugs have different modes of action and are effective in different patient populations; however, there are no ideal biomarkers to determine which patients are optimal candidates for each therapy^2^. The Cancer Genome Atlas (TCGA) data have provided the detailed genomic features of patients with renal cell carcinoma (RCC) by analyzing tumor tissues^3^. For instance, in addition to the loss of chromosome arm 3p encoding tumor suppressor gene *VHL* in more than 70% of ccRCC tumor, other chromosome arm 3p genes *PBRM1*, *SETD2* and *BAP1* were also commonly found in these tumors^3^.

However, the most critical drawback of TCGA data is that the dataset is mainly derived from patients with localized RCC. Numerous studies suggest that genomic evolution may occur during disease progression from localized to metastatic tumor and by selective pressure from different lines of therapy^4–7^.

Since it is difficult to obtain serial tumor tissues during disease progression from the same patients, circulating tumor DNA (ctDNA) derived from blood is an attractive platform to noninvasively identify the temporal evolution of genomic profiles. ctDNA is circulating cell-free DNA (cfDNA) derived from tumor cells and has a length of approximately 150 base pairs, which constitutes 0.1–10% of all cfDNA in blood. The predictive role of ctDNA in non-small cell lung and colorectal cancers has been well-established with longitudinal assessments of genomic profiles. In patients with localized RCC, studies have shown that ctDNA has the potential to serve as a surrogate marker for disease recurrence. Although there are many promising roles of ctDNA in patients with mRCC who are treated with various types of systemic therapy (e.g., ICB), the use of ctDNA in the metastatic setting is still at its infancy.

Here, we aimed to establish the feasibility of ctDNA detection by targeted deep sequencing and explore the predictive role of ctDNA in patients with mRCC who were treated with ICB. Our study showed that ctDNA assessment was feasible in patients with RCC. Moreover, based on the levels of ctDNA, the treatment response in patients with mRCC who received ICB could be predicted.

## Results

### Validation of tumor mutation profiling using ctDNA

**Figure 1:**
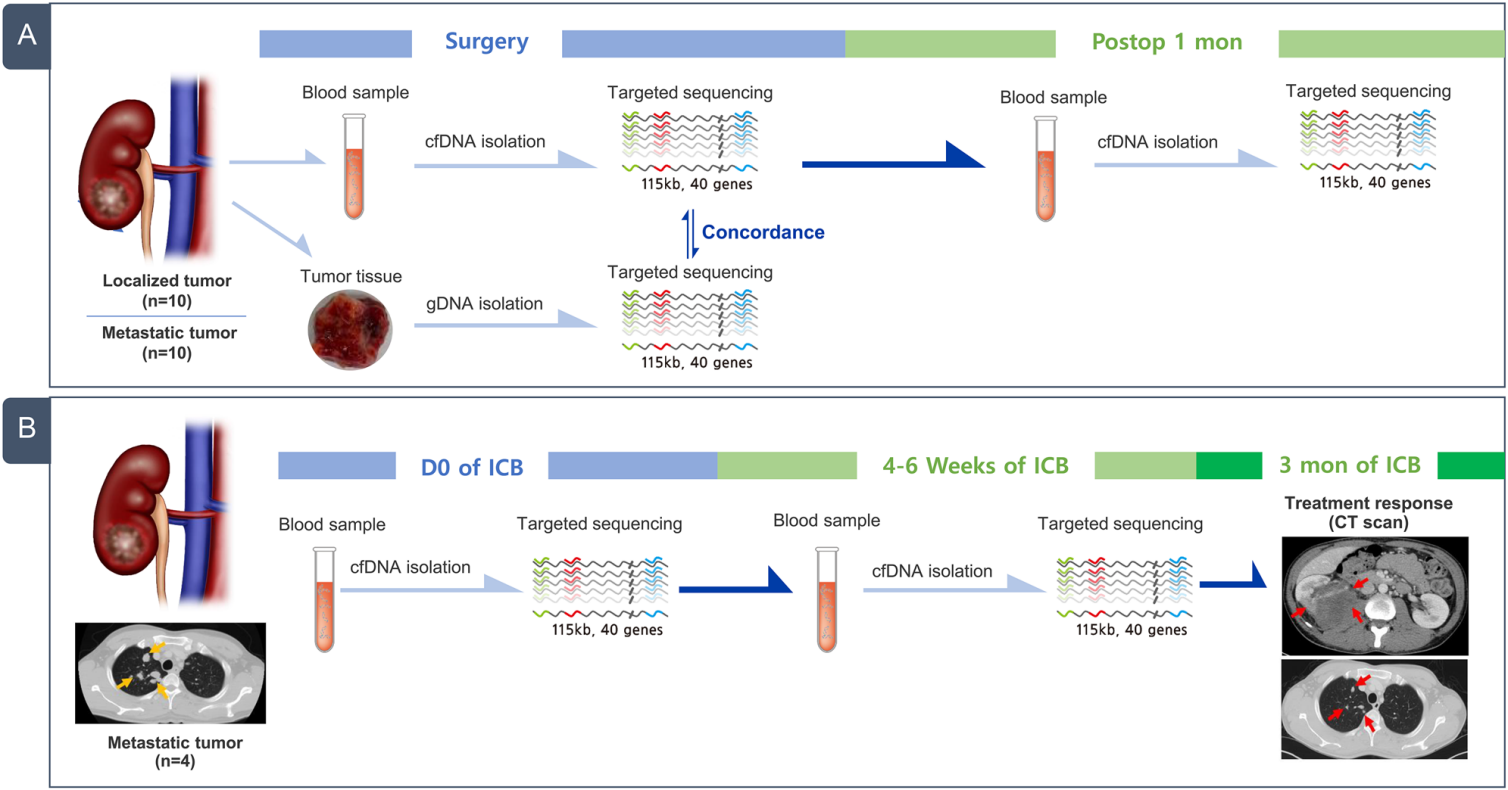
Schematic representation of the current study design. (**A**) Cohort of comparative tumor mutation profiles in tumor samples and circulating tumor DNA (ctDNA) from plasma samples collected from patients with renal cell carcinoma (RCC) who underwent radical nephrectomy (n = 20). (**B**) Cohort of ctDNA examined in patients with metastatic RCC who received first-line immune checkpoint blockade therapy (n = 4).

From the targeted deep sequencing data obtained using the customized panel, we profiled genetic alterations in both plasma and tumor tissues obtained from 20 patients with localized (n = 10) and mRCC (n = 10) who underwent radical nephrectomy (Fig. 1A). The clinicopathological parameters of these patients have been summarized in Table 1. In tumor tissues, somatic mutations within the target gene were detected in 8 of 10 patients with localized RCC (80%) and 7 of 10 patients with mRCC (70%). However, in plasma samples, variants were detected in 4 of 10 patients with localized RCC (40%) and 5 of 10 patients with mRCC (50%). There was no significant difference in the pattern of somatic mutations between localized and metastatic diseases.

**Table 1:** Clinicopathological data of 20 patients with renal cell carcinoma with and without metastasis.

ID	Age	Sex	Tumor size (cm)	pTNM	Metastatic sites	Histology	Nuclear grade	ctDNA detection
P1	66	F	6.5 × 4.5	T3aNxM0	–	Clear cell	II	No
**P2**	**75**	**F**	**8 × 6**	**T2aNxM0**	–	**Clear cell**	**II**	**Yes**
**P3**	**61**	**M**	**6.3 × 5**	**T3aNxM0**	–	**Clear cell**	**II**	**Yes**
P4	36	M	3.4 × 2.5	T1aNxM0	–	Clear cell	III	No
**P5**	**56**	**F**	**5.5 × 5**	**T1bN0M0**	–	**Clear cell**	**III**	**Yes**
**P6**	**62**	**F**	**8.1 × 7.5**	**T3aNxM0**	–	**Clear cell**	**IV**	**Yes**
P7	60	M	7.5 × 5.3	T3aNxM0	–	Clear cell	III	No
P8	68	F	7.5 × 4.5	T3aNxM0	–	Clear cell	IV	No
P9	40	M	6.7 × 5	T1bNxM0	–	Clear cell	II	No
P10	56	M	8.5 × 5.8	T3aNxM0	–	Clear cell	IV	No
**P11**	**58**	**M**	**5 × 4**	**T3aNxM1**	**Lung**	**Clear cell**	**III**	**Yes**
P12	62	M	14 × 13	T3aNxM1	Bone	Clear cell	III	No
P13	64	M	6.7 × 3.5	T4NxM1	Bone	Clear cell and papillary	IV	No
**P14**	**72**	**M**	**17 × 10**	**T3aNxM1**	**Lung**	**Clear cell**	**III**	**Yes**
**P15**	**59**	**M**	**5.5 × 5**	**T3aNxM1**	**Bone**	**Clear cell**	**III**	**Yes**
**P16**	**84**	**F**	**13 × 11.5**	**T3aNxM1**	**Lung**	**Clear cell and papillary**	**III**	**Yes**
P18	44	M	8 × 7	T1bNxM1	Bone	Clear cell	III	No
P19	43	M	4.5 × 3	T2bNxM1	Lung	Clear cell	IV	No
**P20**	**61**	**M**	**12 × 9**	**T3bNxM1**	**Lung, liver, bone**	**Clear cell**	**III**	**Yes**
P21	71	M	14.5 × 8	T3aNxM1	Lung	Clear cell	III	No

P17 was omitted from the study due to follow-up loss after surgery.

Bold marks refer to patients with ctDNA detected in their plasma by targeted deep sequencing.

**Figure 2:**
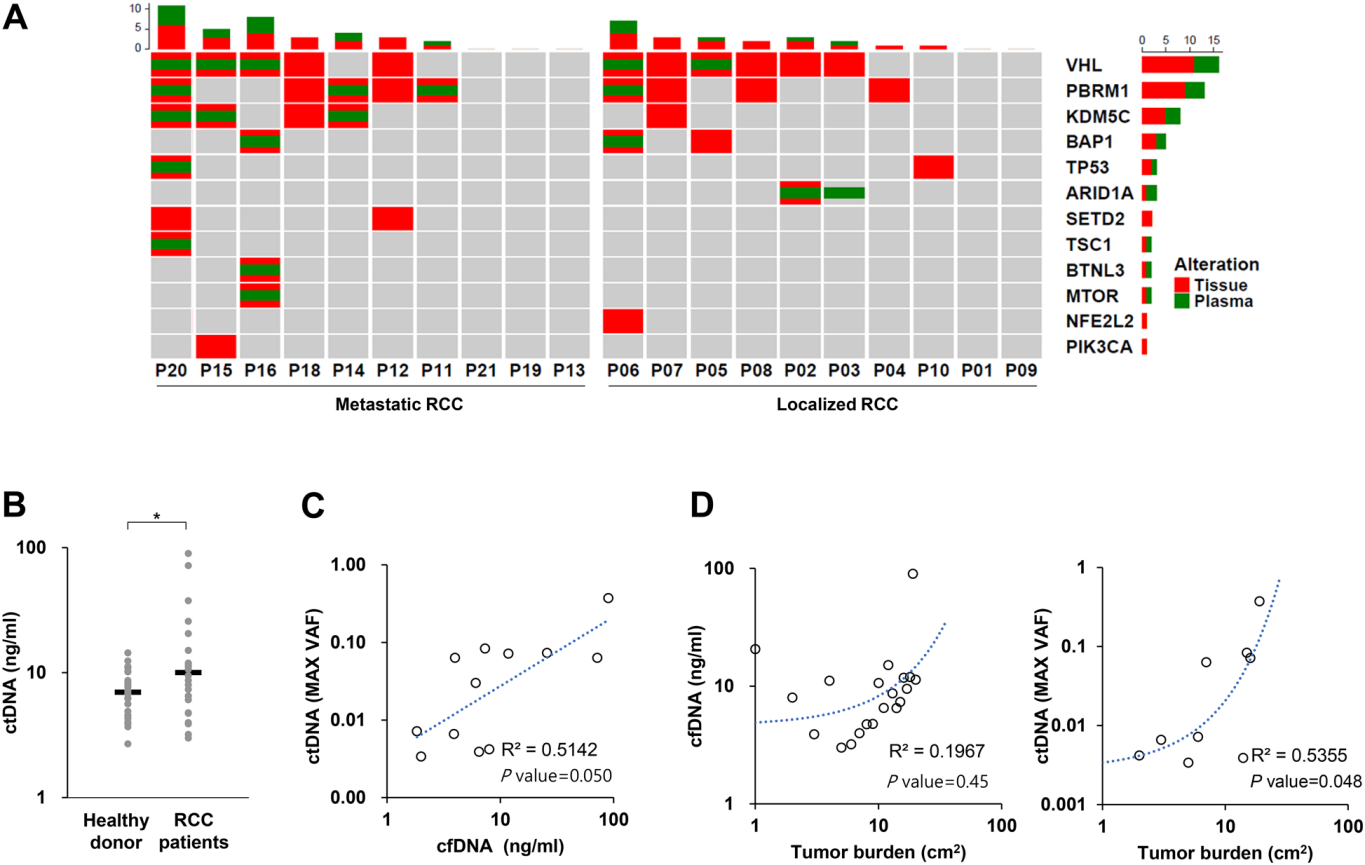
Mutation profiling of tissue and/or plasma samples from patients with renal cell carcinoma (RCC) (**A**) OncoPrint chart shows the occurrence of mutations as profiled by targeted ultra-deep sequencing techniques across 20 patients with RCC. Concordance of variants detected in plasma cell-free DNA (cfDNA) samples compared with biopsy-based sequencing tests is shown in the chart. Each patient sample is indicated as a grey box with mutations indicated in green (plasma) and red (tumor tissue). The barplot is represented as stacked, that show numbers of different varients for each sample and for each gene. (**B**) Distribution of baseline cfDNA levels in patients with RCC and healthy donors. *p value < 0.05. (**c**) The association between the levels of cfDNA and circulating tumor DNA (ctDNA). (**d**) The association between tumor volume and the levels of cfDNA or ctDNA.

Next, we examined the concordance of mutations that were detected in cfDNA samples with those in cancer tissues from these patients. Notably, we found that 53.3% of patients (8/15) who had mutations in the tumor tissues had one or more corresponding mutations in the plasma. Moreover, in 71.4% of patients with metastasis and mutations in tissues, we observed corresponding mutations in plasma samples. Among the 40 RCC-related genes covered by our panel, VHL (25%), PBRM1 (20%), and KDM5C (15%) were highly ranked mutated genes in plasma samples, and 7 of 9 patients (77.7%) harbored at least one mutation in these three genes (Fig. 2A, Supplementary Table S1).

Analysis of the amount of cfDNA in RCC patients revealed that the median value was 9.1 ng/ml (range 3–90), which was significantly higher than that of healthy volunteers (median: 6.9 ng; *p* < 0.026; Fig. 2B). It was confirmed that there was a weak positive correlation between cfDNA and ctDNA (R^2^ coefficient = 0.51; *p* = 0.05; Fig. 2C).

### ctDNA as a surrogate marker of tumor burden in RCC patients

We analyzed whether there was a change in the amount of cfDNA and ctDNA depending on the tumor burden (or diameter) of the primary site as measured by abdomen-pelvis computed tomography (CT) scans at the time of diagnosis. While there was no strong correlation between cfDNA and tumor burden (R^2^ coefficient = 0.20), we found that there was a positive correlation between ctDNA and tumor burden (R^2^ coefficient = 0.53; *p* = 0.048; Fig. 2D).

**Figure 3:**
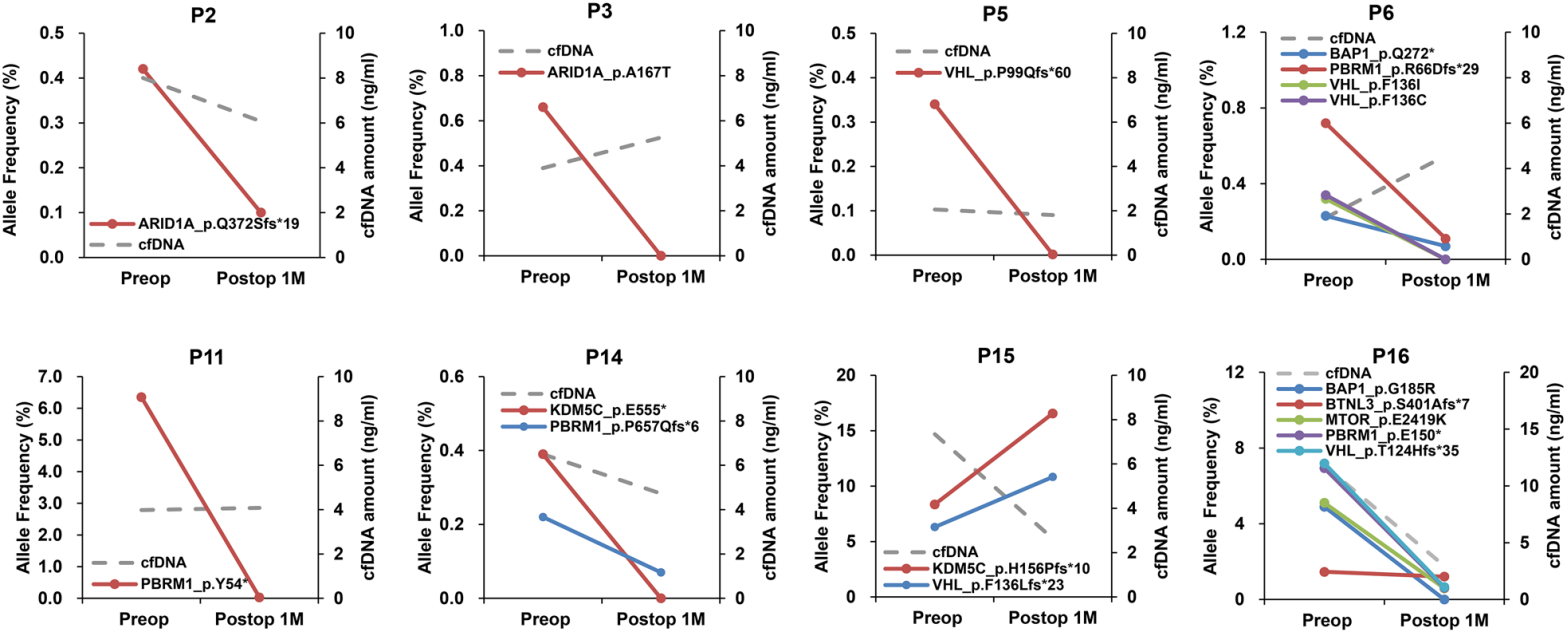
Post-operative circulating tumor DNA (ctDNA) dynamics in patients with renal cell carcinoma (RCC) who underwent radical nephrectomy. Patient blood samples were collected before and 1 month after radical nephrectomy; the ctDNA diversities and levels as assessed by targeted deep sequencing are indicated on the y-axis.

Next, to explore the predictive value of ctDNA in patients with RCC, we analyzed blood samples collected from patients with either localized (n = 10) or mRCC (n = 10) before and 1 month after radical nephrectomy. Among these samples, ctDNA with at least one variant was detected in nine patient samples, among which eight were subjected to further analysis. There was no specific difference in ctDNA variation between localized and mRCC in post-operative samples. Notably, all mutations decreased below 0.1% after surgery in 6 of 8 patients (P2, P3, P5, P6, P11, and P14). However, there were two interesting cases in mRCC (P15 and P16) with respect to ctDNA dynamics. In the case of P15, ctDNA levels significantly increased after cytoreductive nephrectomy compared to the baseline (Fig. 3). We observed that this patient showed an intrinsic resistance to first-line sunitinib treatment and rapid disease progression within 3 months of systemic therapy (Fig. 3). In case of P16, VHL p.T124Hfs*35, PBRM1 p.E150*, BAP1 p.G185R, and MTOR p.E2419K mutations decreased below 0.5%, while BTNL p.S401Afs*7 mutation remained at a low level at 1 month after cytoreductive nephrectomy (Fig. 3). This patient exhibited stable disease (SD) as the best response and no disease progression until 13 months after first-line pazopanib treatment.

### Early prediction of the efficacy of ICB therapy based on ctDNA levels

**Figure 4:**
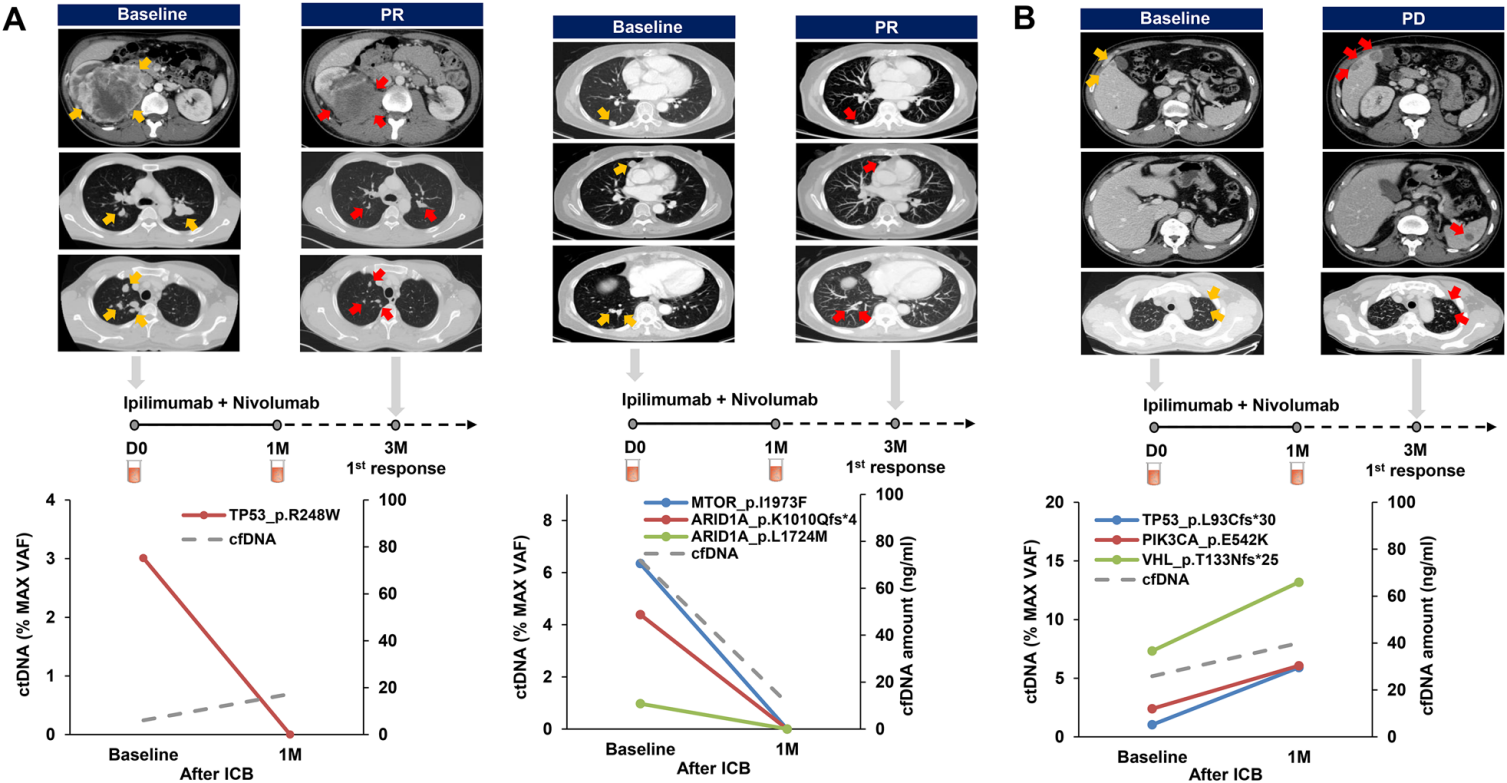
Monitoring therapeutic responses to first-line immune checkpoint blockades (ICB) in patients with metastatic renal cell carcinoma (mRCC). The levels of circulating tumor DNA (ctDNA) were estimated in patients before and after 4–6 weeks of first-line ICB treatment. ICB agents and therapeutic responses are described at the top of the graph for each patient. The therapeutic responsiveness was determined after 3 months of treatment based on abdomen-pelvis and chest CT scans. (**a**) Two patients were classified as showing partial response (PR). (**b**) One patient was classified as showing progressive disease (PD) according to the iRECIST criteria.

To determine the role of ctDNA as an early predictor of responsiveness to ICB treatments, we assessed ctDNA levels in the plasma before and after 4–6 weeks of first-line ipilimumab and nivolumab administration in four patients with mRCC (Fig. 1B). During the treatment course of these patients, therapeutic responses were evaluated based on abdomen-pelvis and chest CT scans that were performed at the baseline and 3 months after ICB treatment. Among the four patients, ctDNA was detected in three patients (75%) before ICB treatment. Interestingly, ctDNA level significantly decreased after ICB treatment in two patients, and they showed partial response (PR) during the assessment of treatment responses after 3 months of ICB therapy (Fig. 4A). One patient had TP53 mutation, while the other had MTOR and ARID1A mutations. In contrast, the levels of ctDNA and TP53, VHL, and PIK3CA gene variants increased after ICB treatment in one patient, and this patient showed progressive disease (PD) during response assessment after 3 months of ICB administration (Fig. 4B).

## Discussion

Precision oncology, such as genome-guided treatment selection, is the most promising approach to treat advanced cancer, and it also provides numerous opportunities for mRCC treatment^8^. However, accurate tumor profiling through sequential treatments for mRCC is currently limited due to the lack of optimal predictive biomarkers. Although the evaluation of ctDNA levels is emerging as a potential alternative method for predicting disease recurrence and treatment responsiveness in RCC, only limited studies have examined the role of ctDNA in RCC.

Yamamoto and colleagues performed targeted sequencing and reported the detection of ctDNA in 30% of 53 patients with clear cell RCC^9^. Among 53 patients, 14 patients were pretreatment status without metastasis, 13 were pretreatment status with metastasis, and 26 were post‐treatment status with metastatic diseases^9^. Of note, the authors found that ctDNA status and fragment size were significantly associated with progression-free survival (PFS) and cancer-specific survival. These results suggested that mutation status and fragmentation of ctDNA can be used as a prognostic marker in patients with RCC^9^. More recently, a research group in UK comprehensively characterized ctDNA in patients with a range of kidney tumor and reported the ctDNA detection rate as 30–40% of overall kidney tumors^10^. In this study, 91 patients with RCC were enrolled as the DIAMOND and MonReC cohorts, respectively. In the DIAMOND cohort, 17 were earlier stage tumor (pT1–2) and 22 were advanced stage tumor (pT3–4), and the MonReC cohort consisted mostly of patients with metastasis^10^. Interestingly, the detection rate of ctDNA was only 35.4% even in patients with metastatic tumors, indicating that ctDNA fractions were lower in RCC compared to other types of malignancies^10^. In contrast to these studies, Pal et al. used a panel comprising 73 genes and showed that genomic alteration was present in 78.6% of 220 consecutive patients with mRCC^4^. In the present study, while ctDNA in the plasma was detected in 45% of patients with RCC among the total population, the detection rate of ctDNA increased up to 71.4% in patients with mRCC. Therefore, ctDNA assessment can be more useful in patients with metastatic tumors but not with localized tumors.

Interestingly, one patient with mRCC (P15) showed increased ctDNA levels after 1 month of surgery compared to the levels at baseline, and we found that this patient had an intrinsic resistance to anti-VEGFR inhibitor therapy. Conversely, three patients with decreased ctDNA levels 1 month after surgery achieved either SD or PR to systemic therapy. Osumi et al. evaluated the predictive role of ctDNA dynamics in patients with metastatic colorectal cancer who were treated with second-line chemotherapy^11^. Patients who had lower changes in ctDNA levels from the baseline, 2 weeks after chemotherapy, showed significantly longer PFS than those with higher ctDNA level changes, indicating that early dynamics in ctDNA levels can be a promising predictor of therapeutic response in these patients^11^. Hrebien and colleagues reported that patients with lower ctDNA levels at 4 weeks after systemic therapy had longer PFS than those with advanced metastatic breast cancer with higher ctDNA levels^12^. Therefore, during the early phases of treatment, ctDNA dynamics can be used as an early predictor for either drug responsiveness or resistance in patients with mRCC receiving systemic therapy.

Recently, clinical trials have revealed that the efficacy of combination therapy with ICB is superior to tyrosine kinase inhibitors alone, and therefore, combination therapy with ICB is being regarded as a first-line treatment option for patients with mRCC^13^. However, there are no optimal biomarkers to predict the responsiveness of ICB therapy. Since ctDNA can reflect the tumor burden and act as a surrogate marker for tumor mutation burden (TMB), it has the potential to serve as a novel predictive biomarker for therapeutic response to ICB^14,15^. Lee et al. investigated whether ctDNA levels at pre-treatment and early treatment could predict the response of ICB in patients with metastatic melanoma^16^. Patients with undetectable ctDNA at either the baseline or within 12 weeks of treatment showed significantly higher response rates (72% and 77%, respectively) than those with elevated ctDNA levels at the baseline and persistently elevated levels during therapy (6%)^16^. Wang and colleagues reported that high TMB estimated by the blood levels of ctDNA indicated better PFS and was associated with higher objective response rates than low blood TMB in patients with non-small cell lung cancer who received anti-PD1 and anti-PD-L1 therapy^14^.

Consistent with the findings of these studies, our results showed that ctDNA levels increased after 1 month of ICB treatment in one patient who showed intrinsic resistance, but ctDNA levels significantly decreased after 1 month of ICB treatment in two patients who showed PR after 3 months of ICB treatment. Although our data were obtained from only three mRCC patients who received ICB therapy, we believe that ctDNA can serve as a potential biomarker for early prediction of ICB responsiveness in patients with mRCC. In a study conducted by Anagnostou et al. early ctDNA elimination was a key prognosticator for better survival outcomes in patients with metastatic lung cancer who received anti-PD1 therapy, and the researchers highlighted that they could predict treatment response, on an average, approximately 9 weeks earlier than radiographic assessment^17^. Raja and colleagues also showed that patients with reduced ctDNA variant allele frequency at 6 weeks after anti-PD-L1 therapy had greater tumor shrinkage with longer survival outcomes^18^. Therefore, we believe that the assessment of ctDNA dynamics should be performed at earlier time points, such as 1 or 2 weeks after ICB administration, to improve the predictive value of ctDNA for early therapeutic decisions.

The present study has a few limitations. First, the sample size was small, which is the most critical drawback of our study. Second, due to a relatively small size of our panel, we could not calculate the accurate TMB in patients with mRCC who received ICB therapy. Third, while the occurrence of clonal evolution due to selective pressure is another key issue in evaluating the role of ctDNA in patients who receive systemic therapy, we could not trace the clonal evolution at different time points in our study.

## Conclusions

In summary, we showed that ctDNA detection in plasma by targeted deep sequencing was feasible in patients with either localized or mRCC. Moreover, the dynamics of ctDNA levels was associated with the therapeutic response of patients with mRCC who were treated with first-line anti-PD1 and anti-CTLA4 combination therapies. Our study provides valuable insights into the promising role of ctDNA as an early predictor of treatment responses in mRCC patients receiving first-line ICB treatment, thereby suggesting a potential strategy for precision oncology.

## Materials and methods

### Patient samples and study design

We performed the current study in two different phases: feasibility and validation tests (Fig. 1). Feasibility test phase included patients with RCC who underwent radical nephrectomy, while validation test phase included patients with mRCC who received first-line ICB therapy. The institutional review board at the Samsung Medical Center approved this study (IRB number: SMC 2018-04-130), and all methods in the current study were conducted in accordance with the Declaration of Helsinki guidelines. A total of 48 peripheral blood samples were prospectively obtained from 24 patients with RCC from November 2018 to February 2020 at our institution. Among the 24 patients, 10 patients had localized RCC, 10 patients had metastatic disease, and 4 patients, who had mRCC, were treated with ICB. We obtained written informed consents from all enrolled patients and removed any personal identifiers by anonymized processing.

The blood samples were collected before and 1 month after radical nephrectomy (n = 20; feasibility test) and before and at 4–6 weeks after ICB treatment (n = 4; validation test). Immediately after surgery, tumor and matched normal tissue specimens (n = 10) were collected and snap frozen for storage. We also assessed clinicopathological variables, such as age at initial diagnosis, sex, primary tumor size as measured by abdomen-pelvis computed tomography (CT) scan, pathological staging according to the 8th edition of the American Joint Committee on Cancer TNM system^19^, histological subtype, Fuhrman nuclear grade, and metastatic sites. Tumor burden was calculated by bi-dimensional measurement (= long axis × short axis) in primary tumor, which were determined by dedicated genitourinary radiologists at our hospital.

Patients were usually followed up for medical history, physical examinations, and routine laboratory tests and imaging, including abdomen-pelvis and chest CT scans, after 3–6 months of surgery. For patients with mRCC treated with ICB, we determined the therapeutic response after every 3 months of treatment by abdomen-pelvis and chest CT scans. The responses were classified as complete response (CR), partial response (PR), stable disease (SD), or progressive disease (PD) according to the iRECIST criteria^20^.

### Sample preparation and cfDNA extraction

Whole blood samples were collected in Cell-Free DNA™ BCT tubes (Streck Inc., Omaha, NE, USA). Plasma was prepared by centrifuging the samples three times with increasing centrifugal force: 840×*g* for 10 min, 1040×*g* for 10 min, and then 5000×*g* for 10 min at room temperature. After separation of plasma in the initial centrifugation step, peripheral blood leukocytes (PBLs) were isolated using RBC Lysis Solution (Qiagen, Santa Clarita, CA, USA). Genomic DNA (gDNA) was isolated from agranulocytes using the QIAamp DNA Mini Kit (Qiagen, Santa Clarita, CA, USA). Plasma DNA was obtained from 2 to 5 ml of plasma using the QIAamp Circulating Nucleic Acid Kit (Qiagen). The AllPrep DNA/RNA Mini Kit (Qiagen) was used to purify gDNA from tissue samples. DNA concentration and purity were measured using a Qubit 2.0 Fluorometer (Life Technologies, Grand Island, NY, USA). The fragment size distribution was measured using the 2200 TapeStation Instrument (Agilent Technologies, Santa Clara, CA, USA). The amount of cfDNA in healthy volunteers has been analyzed in our previous study on lymphoma biomarkers^21^.

### Library preparation

Purified gDNA was sonicated (7 min, 0.5% duty, intensity of 0.1, and 50 cycles/burst) into 150–200 bp fragments using a Covaris S2 sonicator (Covaris Inc. Woburn, MA, USA). To construct reference libraries, tissue samples were subjected to targeted sequencing using tissues that were previously acquired for diagnosis. The tumor sample libraries were constructed using the SureSelect XT Reagent kit, HSQ (Agilent Technologies), according to the manufacturer’s instructions. The PBLs and plasma DNA libraries were created using the KAPA Hyper Prep Kit (Kapa Biosystems, Woburn, MA, USA). Briefly, we performed end repair and A-tailing according to the manufacturer’s protocol, followed by adaptor ligation at 4 °C overnight using a pre-indexed PentAdapter™ (PentaBase ApS, Denmark). For the library construction of biopsy specimens, hybrid selection was performed using customized baits targeting 40 RCC-related genes (LiquidSCAN RCC panel, GENINUS, Korea). To achieve a mean sequencing depth of approximately 10,000× prior to duplicate removal, we designed a pool of RNA baits targeting 40 RCC-associated genes, including hotspot mutations (Table 2).

**Table 2:** List of genes for targeted deep sequencing.

APC	CCNB2	KDM5C	MTOR	NRAS	RHEB	TSC1
ARID1A	EGFR	KIT	MYC	OPTC	SETD2	TXNIP
BAP1	ERBB2	KLRC2	NF1	OR2L8	SLITRK6	VHL
BRAF	ERBB4	MAX	NFE2L2	PBRM1	SPG21	ZNF91
BRCC3	FGFR2	MET	NOTCH1	PIK3CA	TERT_promoter	
BTNL3	GNA13	MSR1	NPNT	PTEN	TP53	

### Analysis of sequencing data

All data were aligned to the hg19 reference genome using BWA-mem (v0.7.5; Wellcome Trust Sanger Institute, Cambridge, UK). We created custom-made Python (v2.7.9; Python Software Foundation, Delaware, United States) scripts to process the duplicate reads. We modified iDES methods and created the scripts^22^. GATK (v4.0.0; Broad Institute, Cambridge, UK)^23^, Picard (v2.9.4; Broad Institute, Cambridge, UK), and SAMTOOLS (v1.6; Wellcome Trust Sanger Institute, Cambridge, UK)^24^ were used for base quality recalibration, cross-validation of UID family, and sorting of SAM and BAM files, respectively. During processing, discordant pairs and off-target reads were filtered out. The filtering steps to identify the variants were performed as described previously^25^. The quantitative levels of ctDNA were measured as genome equivalents that were determined as the product of total cfDNA concentration and the maximal allele fraction of somatic mutations.

OncoPrint generated by the Complex Heatmaps package (http://bioconductor.org/packages/release/bioc/html/ComplexHeatmap.html) in R 3.5.0 software^26^.

### Statistical analysis

The correlation between tumor burden and the amount of DNA was calculated using the Pearson correlation coefficient. Descriptive statistics were determined as proportions and medians, and the intergroup comparisons for categorical variables were assessed by Fisher’s exact test. Statistical analysis was performed using R 3.4.2, where *p* values < 0.05 were considered significant.

## Supplementary Information

Supplementary Information
