# Supplementary material for: Potential of circulating tumor DNA as a predictor of therapeutic responses to immune checkpoint blockades in metastatic renal cell carcinoma
Source: Sci Rep. 2021 Mar 10;11:5600. doi: 10.1038/s41598-021-85099-4 (PMC7970914; doi:10.1038/s41598-021-85099-4)
Supplement: Supplementary file 1 — Supplementary Information [file 41598_2021_85099_MOESM1_ESM.docx]

**Title**

Potential of circulating tumor DNA as a predictor of therapeutic responses to immune checkpoint blockades in metastatic renal cell carcinoma

**Authors and institutions**

Yeon Jeong Kim^1^, Yumi Kang^2^, Hyun Hwan Sung^2^, Hwang Gyun Jeon^2^, Byong Chang Jeong^2^, Seong Il Seo^2^, Seong Soo Jeon^2^, Hyun Moo Lee^2^, Donghyun Park^3^, Woong-Yang Park^1,4^ and Minyong Kang^2,5,6,*^

^1^Samsung Genome Institute, Samsung Medical Center; ^2^Department of Urology, Samsung Medical Center, Sungkyunkwan University School of Medicine; ^3^Geninus, Seoul; ^4^Department of Molecular Cell Biology, Sungkyunkwan University School of Medicine, Suwon; ^5^Department of Health Sciences and Technology; ^6^Department of Digital Health, SAIHST, Sungkyunkwan University, Seoul, Republic of Korea

**Running title**: ctDNA and ICB responsiveness in mRCC

**Word count**: Abstract (200), Text (3,104)

***Corresponding author**

Minyong Kang, MD, PhD

Department of Urology, Samsung Medical Center, Sungkyunkwan University School of Medicine; Department of Health Sciences and Technology; Department of Digital Health, SAIHST, Sungkyunkwan University

81 Irwon-ro, Gangnam-gu, Seoul 06351, South Korea
Tel: +82-2-3410-1138, Fax: +82-2-3410-6992

E-mail: [m79.kang@skku.edu](mailto:m79.kang@skku.edu)

**Table S1: Summary of sequencing metrics**

| Patient ID | Sample ID | Chr | Start | End | Ref | Alt | RefSeq | Gene | Func | Amino acid change | cDNA position | Count | Coverage | VAF |
| --- | --- | --- | --- | --- | --- | --- | --- | --- | --- | --- | --- | --- | --- | --- |
| P10 | PBL | chr17 | 7578275 | 7578275 | G | A | NM_000546 | TP53 | stop_gained | p.Q192* | c.574C>T | 0 | 4686 | 0.00% |
| P10 | Plasma1 | chr17 | 7578275 | 7578275 | G | A | NM_000546 | TP53 | stop_gained | p.Q192* | c.574C>T | 0 | 4530 | 0.00% |
| P10 | Tissue | chr17 | 7578275 | 7578275 | G | A | NM_000546 | TP53 | stop_gained | p.Q192* | c.574C>T | 619 | 3991 | 15.51% |
| P2 | Tissue | chr1 | 27024002 | 27024002 | G | - | NM_006015 | ARID1A | frameshift_variant | p.Q372Sfs*19 | c.1113del | 1059 | 4292 | 24.67% |
| P2 | Plasma1 | chr1 | 27024002 | 27024002 | G | - | NM_006015 | ARID1A | frameshift_variant | p.Q372Sfs*19 | c.1113del | 14 | 3339 | 0.42% |
| P2 | Plasma2 | chr1 | 27024002 | 27024002 | G | - | NM_006015 | ARID1A | frameshift_variant | p.Q372Sfs*19 | c.1113del | 1 | 968 | 0.10% |
| P2 | PBL | chr1 | 27024002 | 27024002 | G | - | NM_006015 | ARID1A | frameshift_variant | p.Q372Sfs*19 | c.1113del | 13 | 3399 | 0.38% |
| P2 | Tissue | chr3 | 10191553 | 10191553 | - | A | NM_000551 | VHL | frameshift_variant | p.S183Ifs*73 | c.546_547insA | 2042 | 5031 | 40.59% |
| P2 | Plasma1 | chr3 | 10191553 | 10191553 | - | A | NM_000551 | VHL | frameshift_variant | p.S183Ifs*73 | c.546_547insA | 15 | 9765 | 0.15% |
| P2 | Plasma2 | chr3 | 10191553 | 10191553 | - | A | NM_000551 | VHL | frameshift_variant | p.S183Ifs*73 | c.546_547insA | 0 | 4564 | 0.00% |
| P2 | PBL | chr3 | 10191553 | 10191553 | - | A | NM_000551 | VHL | frameshift_variant | p.S183Ifs*73 | c.546_547insA | 6 | 7073 | 0.08% |
| P2 | PBL | chr3 | 52651478 | 52651478 | T | C | NM_181042 | PBRM1 | missense_variant | p.R540G | c.1618A>G | 0 | 5246 | 0.00% |
| P2 | Plasma1 | chr3 | 52651478 | 52651478 | T | C | NM_181042 | PBRM1 | missense_variant | p.R540G | c.1618A>G | 0 | 5719 | 0.00% |
| P2 | Tissue | chr3 | 52651478 | 52651478 | T | C | NM_181042 | PBRM1 | missense_variant | p.R540G | c.1618A>G | 1023 | 3124 | 32.75% |
| P2 | Plasma2 | chr3 | 52651478 | 52651478 | T | C | NM_181042 | PBRM1 | missense_variant | p.R540G | c.1618A>G | 0 | 2895 | 0.00% |
| P3 | PBL | chr1 | 27023393 | 27023393 | G | A | NM_006015 | ARID1A | missense_variant | p.A167T | c.499G>A | 0 | 1763 | 0.00% |
| P3 | Plasma1 | chr1 | 27023393 | 27023393 | G | A | NM_006015 | ARID1A | missense_variant | p.A167T | c.499G>A | 7 | 1054 | 0.66% |
| P3 | Tissue | chr1 | 27023393 | 27023393 | G | A | NM_006015 | ARID1A | missense_variant | p.A167T | c.499G>A | 0 | 1628 | 0.00% |
| P3 | Plasma2 | chr1 | 27023393 | 27023393 | G | A | NM_006015 | ARID1A | missense_variant | p.A167T | c.499G>A | 0 | 200 | 0.00% |
| P3 | Tissue | chr3 | 10183693 | 10183693 | - | G | NM_000551 | VHL | frameshift_variant | p.E55Gfs*77 | c.163dup | 1511 | 3448 | 43.82% |
| P3 | Plasma1 | chr3 | 10183693 | 10183693 | - | G | NM_000551 | VHL | frameshift_variant | p.E55Gfs*77 | c.163dup | 1 | 2719 | 0.04% |
| P3 | Plasma2 | chr3 | 10183693 | 10183693 | - | G | NM_000551 | VHL | frameshift_variant | p.E55Gfs*77 | c.163dup | 4 | 1072 | 0.37% |
| P3 | PBL | chr3 | 10183693 | 10183693 | - | G | NM_000551 | VHL | frameshift_variant | p.E55Gfs*77 | c.163dup | 11 | 6874 | 0.16% |
| P4 | Tissue | chr3 | 52588796 | 52588796 | G | - | NM_018313 | PBRM1 | frameshift_variant | p.P1411Lfs*21 | c.4232del | 516 | 6652 | 7.76% |
| P4 | Plasma1 | chr3 | 52588796 | 52588796 | G | - | NM_018313 | PBRM1 | frameshift_variant | p.P1411Lfs*21 | c.4232del | 15 | 9184 | 0.16% |
| P4 | PBL | chr3 | 52588796 | 52588796 | G | - | NM_018313 | PBRM1 | frameshift_variant | p.P1411Lfs*21 | c.4232del | 4 | 6671 | 0.06% |
| P5 | Tissue | chr3 | 10183825 | 10183825 | C | - | NM_000551 | VHL | frameshift_variant | p.P99Qfs*60 | c.296del | 1213 | 5848 | 20.74% |
| P5 | Plasma1 | chr3 | 10183825 | 10183825 | C | - | NM_000551 | VHL | frameshift_variant | p.P99Qfs*60 | c.296del | 16 | 4755 | 0.34% |
| P5 | Plasma2 | chr3 | 10183825 | 10183825 | C | - | NM_000551 | VHL | frameshift_variant | p.P99Qfs*60 | c.296del | 2 | 1577 | 0.13% |
| P5 | PBL | chr3 | 10183825 | 10183825 | C | - | NM_000551 | VHL | frameshift_variant | p.P99Qfs*60 | c.296del | 7 | 4590 | 0.15% |
| P5 | Tissue | chr3 | 52436435 | 52436438 | TGCC | - | NM_004656 | BAP1 | splice_acceptor_variant,coding_sequence_variant | NA | c.2057-1_2059del | 1035 | 6998 | 14.79% |
| P5 | Plasma1 | chr3 | 52436435 | 52436438 | TGCC | - | NM_004656 | BAP1 | splice_acceptor_variant,coding_sequence_variant | NA | c.2057-1_2059del | 1 | 7385 | 0.01% |
| P5 | Plasma2 | chr3 | 52436435 | 52436438 | TGCC | - | NM_004656 | BAP1 | splice_acceptor_variant,coding_sequence_variant | NA | c.2057-1_2059del | 1 | 3133 | 0.03% |
| P5 | PBL | chr3 | 52436435 | 52436438 | TGCC | - | NM_004656 | BAP1 | splice_acceptor_variant,coding_sequence_variant | NA | c.2057-1_2059del | 6 | 5408 | 0.11% |
| P6 | PBL | chr2 | 178095999 | 1.78E+08 | G | T | NM_006164 | NFE2L2 | missense_variant | p.D444E | c.1332C>A | 1 | 5293 | 0.02% |
| P6 | Plasma1 | chr2 | 178095999 | 1.78E+08 | G | T | NM_006164 | NFE2L2 | missense_variant | p.D444E | c.1332C>A | 3 | 5486 | 0.05% |
| P6 | Tissue | chr2 | 178095999 | 1.78E+08 | G | T | NM_006164 | NFE2L2 | missense_variant | p.D444E | c.1332C>A | 462 | 5983 | 7.72% |
| P6 | Plasma2 | chr2 | 178095999 | 1.78E+08 | G | T | NM_006164 | NFE2L2 | missense_variant | p.D444E | c.1332C>A | 0 | 2605 | 0.00% |
| P6 | PBL | chr3 | 10188263 | 10188263 | T | A | NM_000551 | VHL | missense_variant | p.F136I | c.406T>A | 0 | 4432 | 0.00% |
| P6 | Plasma1 | chr3 | 10188263 | 10188263 | T | A | NM_000551 | VHL | missense_variant | p.F136I | c.406T>A | 17 | 5246 | 0.32% |
| P6 | Tissue | chr3 | 10188263 | 10188263 | T | A | NM_000551 | VHL | missense_variant | p.F136I | c.406T>A | 1932 | 3495 | 55.28% |
| P6 | Plasma2 | chr3 | 10188263 | 10188263 | T | A | NM_000551 | VHL | missense_variant | p.F136I | c.406T>A | 0 | 2357 | 0.00% |
| P6 | PBL | chr3 | 10188264 | 10188264 | T | G | NM_000551 | VHL | missense_variant | p.F136C | c.407T>G | 0 | 4446 | 0.00% |
| P6 | Plasma1 | chr3 | 10188264 | 10188264 | T | G | NM_000551 | VHL | missense_variant | p.F136C | c.407T>G | 18 | 5237 | 0.34% |
| P6 | Tissue | chr3 | 10188264 | 10188264 | T | G | NM_000551 | VHL | missense_variant | p.F136C | c.407T>G | 1884 | 3467 | 54.34% |
| P6 | Plasma2 | chr3 | 10188264 | 10188264 | T | G | NM_000551 | VHL | missense_variant | p.F136C | c.407T>G | 0 | 2351 | 0.00% |
| P6 | PBL | chr3 | 52439898 | 52439898 | G | A | NM_004656 | BAP1 | stop_gained | p.Q272* | c.814C>T | 0 | 4085 | 0.00% |
| P6 | Plasma1 | chr3 | 52439898 | 52439898 | G | A | NM_004656 | BAP1 | stop_gained | p.Q272* | c.814C>T | 13 | 5535 | 0.23% |
| P6 | Tissue | chr3 | 52439898 | 52439898 | G | A | NM_004656 | BAP1 | stop_gained | p.Q272* | c.814C>T | 1928 | 3563 | 54.11% |
| P6 | Plasma2 | chr3 | 52439898 | 52439898 | G | A | NM_004656 | BAP1 | stop_gained | p.Q272* | c.814C>T | 2 | 2781 | 0.07% |
| P6 | Tissue | chr3 | 52712557 | 52712557 | G | - | NM_181042 | PBRM1 | frameshift_variant | p.R66Dfs*29 | c.195del | 2536 | 5166 | 49.09% |
| P6 | Plasma1 | chr3 | 52712557 | 52712557 | G | - | NM_181042 | PBRM1 | frameshift_variant | p.R66Dfs*29 | c.195del | 75 | 10450 | 0.72% |
| P6 | Plasma2 | chr3 | 52712557 | 52712557 | G | - | NM_181042 | PBRM1 | frameshift_variant | p.R66Dfs*29 | c.195del | 5 | 4729 | 0.11% |
| P6 | PBL | chr3 | 52712557 | 52712557 | G | - | NM_181042 | PBRM1 | frameshift_variant | p.R66Dfs*29 | c.195del | 5 | 6190 | 0.08% |
| P7 | PBL | chr3 | 10183833 | 10183833 | T | C | NM_000551 | VHL | missense_variant | p.L101P | c.302T>C | 0 | 3520 | 0.00% |
| P7 | Plasma1 | chr3 | 10183833 | 10183833 | T | C | NM_000551 | VHL | missense_variant | p.L101P | c.302T>C | 0 | 3574 | 0.00% |
| P7 | Tissue | chr3 | 10183833 | 10183833 | T | C | NM_000551 | VHL | missense_variant | p.L101P | c.302T>C | 833 | 3376 | 24.67% |
| P7 | Tissue | chr3 | 52643954 | 52643954 | A | - | NM_181042 | PBRM1 | frameshift_variant | p.S648Lfs*8 | c.1942del | 1540 | 6344 | 24.27% |
| P7 | Plasma1 | chr3 | 52643954 | 52643954 | A | - | NM_181042 | PBRM1 | frameshift_variant | p.S648Lfs*8 | c.1942del | 3 | 9720 | 0.03% |
| P7 | PBL | chr3 | 52643954 | 52643954 | A | - | NM_181042 | PBRM1 | frameshift_variant | p.S648Lfs*8 | c.1942del | 7 | 6064 | 0.12% |
| P7 | Tissue | chrX | 53223015 | 53223016 | TC | - | NM_004187 | KDM5C | frameshift_variant | p.N1353Wfs*8 | c.4056_4057del | 177 | 3172 | 5.58% |
| P7 | Plasma1 | chrX | 53223015 | 53223016 | TC | - | NM_004187 | KDM5C | frameshift_variant | p.N1353Wfs*8 | c.4056_4057del | 0 | 4653 | 0.00% |
| P7 | PBL | chrX | 53223015 | 53223016 | TC | - | NM_004187 | KDM5C | frameshift_variant | p.N1353Wfs*8 | c.4056_4057del | 5 | 2681 | 0.19% |
| P8 | PBL | chr3 | 10188208 | 10188208 | G | A | NM_000551 | VHL | stop_gained | p.W117* | c.351G>A | 2 | 3096 | 0.06% |
| P8 | Plasma1 | chr3 | 10188208 | 10188208 | G | A | NM_000551 | VHL | stop_gained | p.W117* | c.351G>A | 4 | 5797 | 0.07% |
| P8 | Tissue | chr3 | 10188208 | 10188208 | G | A | NM_000551 | VHL | stop_gained | p.W117* | c.351G>A | 885 | 2631 | 33.64% |
| P8 | PBL | chr3 | 52620652 | 52620652 | C | A | NM_181042 | PBRM1 | missense_variant | p.C1059F | c.3176G>T | 2 | 3896 | 0.05% |
| P8 | Plasma1 | chr3 | 52620652 | 52620652 | C | A | NM_181042 | PBRM1 | missense_variant | p.C1059F | c.3176G>T | 0 | 7756 | 0.00% |
| P8 | Tissue | chr3 | 52620652 | 52620652 | C | A | NM_181042 | PBRM1 | missense_variant | p.C1059F | c.3176G>T | 1132 | 3389 | 33.40% |
| P11 | Tissue | chr3 | 52712589 | 52712590 | TA | - | NM_181042 | PBRM1 | stop_gained,frameshift_variant | p.Y54* | c.162_163del | 1803 | 4303 | 41.90% |
| P11 | Plasma1 | chr3 | 52712589 | 52712590 | TA | - | NM_181042 | PBRM1 | stop_gained,frameshift_variant | p.Y54* | c.162_163del | 632 | 9951 | 6.35% |
| P11 | Plasma2 | chr3 | 52712589 | 52712590 | TA | - | NM_181042 | PBRM1 | stop_gained,frameshift_variant | p.Y54* | c.162_163del | 1 | 2959 | 0.03% |
| P11 | PBL | chr3 | 52712589 | 52712590 | TA | - | NM_181042 | PBRM1 | stop_gained,frameshift_variant | p.Y54* | c.162_163del | 8 | 5481 | 0.15% |
| P20 | PBL | chr17 | 7578449 | 7578449 | C | T | NM_000546 | TP53 | missense_variant | p.A161T | c.481G>A | 1 | 3894 | 0.03% |
| P20 | Plasma1 | chr17 | 7578449 | 7578449 | C | T | NM_000546 | TP53 | missense_variant | p.A161T | c.481G>A | 1531 | 5963 | 25.67% |
| P20 | Tissue | chr17 | 7578449 | 7578449 | C | T | NM_000546 | TP53 | missense_variant | p.A161T | c.481G>A | 439 | 4382 | 10.02% |
| P20 | Tissue | chr3 | 10188215 | 10188215 | A | - | NM_000551 | VHL | frameshift_variant | p.R120Efs*39 | c.358del | 1224 | 5341 | 22.92% |
| P20 | Plasma1 | chr3 | 10188215 | 10188215 | A | - | NM_000551 | VHL | frameshift_variant | p.R120Efs*39 | c.358del | 3118 | 12159 | 25.64% |
| P20 | PBL | chr3 | 10188215 | 10188215 | A | - | NM_000551 | VHL | frameshift_variant | p.R120Efs*39 | c.358del | 1 | 4516 | 0.02% |
| P20 | PBL | chr3 | 47165515 | 47165515 | G | C | NM_014159 | SETD2 | stop_gained | p.S204* | c.611C>G | 0 | 4287 | 0.00% |
| P20 | Plasma1 | chr3 | 47165515 | 47165515 | G | C | NM_014159 | SETD2 | stop_gained | p.S204* | c.611C>G | 0 | 5673 | 0.00% |
| P20 | Tissue | chr3 | 47165515 | 47165515 | G | C | NM_014159 | SETD2 | stop_gained | p.S204* | c.611C>G | 667 | 5210 | 12.80% |
| P20 | PBL | chr3 | 52692298 | 52692298 | G | A | NM_181042 | PBRM1 | stop_gained | p.Q188* | c.562C>T | 0 | 4362 | 0.00% |
| P20 | Plasma1 | chr3 | 52692298 | 52692298 | G | A | NM_181042 | PBRM1 | stop_gained | p.Q188* | c.562C>T | 1645 | 4430 | 37.13% |
| P20 | Tissue | chr3 | 52692298 | 52692298 | G | A | NM_181042 | PBRM1 | stop_gained | p.Q188* | c.562C>T | 1278 | 5082 | 25.15% |
| P20 | Tissue | chr9 | 135782190 | 1.36E+08 | - | G | NM_000368 | TSC1 | frameshift_variant | p.L456Pfs*3 | c.1366dup | 864 | 7614 | 11.35% |
| P20 | Plasma1 | chr9 | 135782190 | 1.36E+08 | - | G | NM_000368 | TSC1 | frameshift_variant | p.L456Pfs*3 | c.1366dup | 4573 | 19055 | 24.00% |
| P20 | PBL | chr9 | 135782190 | 1.36E+08 | - | G | NM_000368 | TSC1 | frameshift_variant | p.L456Pfs*3 | c.1366dup | 4 | 5702 | 0.07% |
| P20 | PBL | chrX | 53254032 | 53254032 | A | G | NM_004187 | KDM5C | missense_variant | p.C14R | c.40T>C | 0 | 1511 | 0.00% |
| P20 | Plasma1 | chrX | 53254032 | 53254032 | A | G | NM_004187 | KDM5C | missense_variant | p.C14R | c.40T>C | 653 | 1747 | 37.38% |
| P20 | Tissue | chrX | 53254032 | 53254032 | A | G | NM_004187 | KDM5C | missense_variant | p.C14R | c.40T>C | 324 | 1995 | 16.24% |
| P12 | Tissue | chr3 | 10188303 | 10188303 | C | - | NM_000551 | VHL | frameshift_variant | p.N150Ifs*9 | c.447del | 1653 | 4819 | 34.30% |
| P12 | Plasma1 | chr3 | 10188303 | 10188303 | C | - | NM_000551 | VHL | frameshift_variant | p.N150Ifs*9 | c.447del | 3 | 7221 | 0.04% |
| P12 | PBL | chr3 | 10188303 | 10188303 | C | - | NM_000551 | VHL | frameshift_variant | p.N150Ifs*9 | c.447del | 5 | 5158 | 0.10% |
| P12 | PBL | chr3 | 47061324 | 47061324 | T | A | NM_014159 | SETD2 | stop_gained | p.K2453* | c.7357A>T | 0 | 4347 | 0.00% |
| P12 | Plasma1 | chr3 | 47061324 | 47061324 | T | A | NM_014159 | SETD2 | stop_gained | p.K2453* | c.7357A>T | 0 | 4961 | 0.00% |
| P12 | Tissue | chr3 | 47061324 | 47061324 | T | A | NM_014159 | SETD2 | stop_gained | p.K2453* | c.7357A>T | 1285 | 3724 | 34.51% |
| P12 | PBL | chr3 | 52702582 | 52702582 | A | C | NM_181042 | PBRM1 | missense_variant | p.Y106D | c.316T>G | 0 | 4616 | 0.00% |
| P12 | Plasma1 | chr3 | 52702582 | 52702582 | A | C | NM_181042 | PBRM1 | missense_variant | p.Y106D | c.316T>G | 0 | 5117 | 0.00% |
| P12 | Tissue | chr3 | 52702582 | 52702582 | A | C | NM_181042 | PBRM1 | missense_variant | p.Y106D | c.316T>G | 1494 | 4133 | 36.15% |
| P14 | Tissue | chr3 | 52643928 | 52643928 | A | - | NM_181042 | PBRM1 | frameshift_variant | p.P657Qfs*6 | c.1968del | 2292 | 6703 | 34.19% |
| P14 | Plasma1 | chr3 | 52643928 | 52643928 | A | - | NM_181042 | PBRM1 | frameshift_variant | p.P657Qfs*6 | c.1968del | 28 | 12969 | 0.22% |
| P14 | Plasma2 | chr3 | 52643928 | 52643928 | A | - | NM_181042 | PBRM1 | frameshift_variant | p.P657Qfs*6 | c.1968del | 2 | 3055 | 0.07% |
| P14 | PBL | chr3 | 52643928 | 52643928 | A | - | NM_181042 | PBRM1 | frameshift_variant | p.P657Qfs*6 | c.1968del | 9 | 7180 | 0.13% |
| P14 | PBL | chrX | 53239679 | 53239679 | C | A | NM_004187 | KDM5C | stop_gained | p.E555* | c.1663G>T | 0 | 2555 | 0.00% |
| P14 | Plasma1 | chrX | 53239679 | 53239679 | C | A | NM_004187 | KDM5C | stop_gained | p.E555* | c.1663G>T | 18 | 4617 | 0.39% |
| P14 | Tissue | chrX | 53239679 | 53239679 | C | A | NM_004187 | KDM5C | stop_gained | p.E555* | c.1663G>T | 1810 | 3283 | 55.13% |
| P14 | Plasma2 | chrX | 53239679 | 53239679 | C | A | NM_004187 | KDM5C | stop_gained | p.E555* | c.1663G>T | 0 | 1102 | 0.00% |
| P15 | Tissue | chr3 | 10188263 | 10188263 | T | - | NM_000551 | VHL | frameshift_variant | p.F136Lfs*23 | c.408del | 1250 | 5995 | 20.85% |
| P15 | Plasma1 | chr3 | 10188263 | 10188263 | T | - | NM_000551 | VHL | frameshift_variant | p.F136Lfs*23 | c.408del | 463 | 7329 | 6.32% |
| P15 | Plasma2 | chr3 | 10188263 | 10188263 | T | - | NM_000551 | VHL | frameshift_variant | p.F136Lfs*23 | c.408del | 296 | 2734 | 10.83% |
| P15 | PBL | chr3 | 10188263 | 10188263 | T | - | NM_000551 | VHL | frameshift_variant | p.F136Lfs*23 | c.408del | 5 | 6427 | 0.08% |
| P15 | PBL | chr3 | 178922324 | 1.79E+08 | G | A | NM_006218 | PIK3CA | missense_variant | p.E365K | c.1093G>A | 2 | 5727 | 0.03% |
| P15 | Plasma1 | chr3 | 178922324 | 1.79E+08 | G | A | NM_006218 | PIK3CA | missense_variant | p.E365K | c.1093G>A | 0 | 5855 | 0.00% |
| P15 | Tissue | chr3 | 178922324 | 1.79E+08 | G | A | NM_006218 | PIK3CA | missense_variant | p.E365K | c.1093G>A | 1057 | 6669 | 15.85% |
| P15 | Plasma2 | chr3 | 178922324 | 1.79E+08 | G | A | NM_006218 | PIK3CA | missense_variant | p.E365K | c.1093G>A | 0 | 1418 | 0.00% |
| P15 | Tissue | chrX | 53247034 | 53247034 | - | G | NM_004187 | KDM5C | frameshift_variant | p.H156Pfs*10 | c.466dup | 83 | 3572 | 2.32% |
| P15 | Plasma1 | chrX | 53247034 | 53247034 | - | G | NM_004187 | KDM5C | frameshift_variant | p.H156Pfs*10 | c.466dup | 353 | 4226 | 8.35% |
| P15 | Plasma2 | chrX | 53247034 | 53247034 | - | G | NM_004187 | KDM5C | frameshift_variant | p.H156Pfs*10 | c.466dup | 336 | 2029 | 16.56% |
| P15 | PBL | chrX | 53247034 | 53247034 | - | G | NM_004187 | KDM5C | frameshift_variant | p.H156Pfs*10 | c.466dup | 11 | 3160 | 0.35% |
| P16 | PBL | chr1 | 11174420 | 11174420 | C | T | NM_004958 | MTOR | missense_variant | p.E2419K | c.7255G>A | 0 | 5001 | 0.00% |
| P16 | Plasma1 | chr1 | 11174420 | 11174420 | C | T | NM_004958 | MTOR | missense_variant | p.E2419K | c.7255G>A | 351 | 6874 | 5.11% |
| P16 | Tissue | chr1 | 11174420 | 11174420 | C | T | NM_004958 | MTOR | missense_variant | p.E2419K | c.7255G>A | 34 | 129 | 26.36% |
| P16 | Plasma2 | chr1 | 11174420 | 11174420 | C | T | NM_004958 | MTOR | missense_variant | p.E2419K | c.7255G>A | 11 | 1858 | 0.59% |
| P16 | Tissue | chr3 | 10188224 | 10188224 | G | - | NM_000551 | VHL | frameshift_variant | p.T124Hfs*35 | c.369del | 70 | 164 | 42.68% |
| P16 | Plasma1 | chr3 | 10188224 | 10188224 | G | - | NM_000551 | VHL | frameshift_variant | p.T124Hfs*35 | c.369del | 775 | 10785 | 7.19% |
| P16 | Plasma2 | chr3 | 10188224 | 10188224 | G | - | NM_000551 | VHL | frameshift_variant | p.T124Hfs*35 | c.369del | 16 | 2437 | 0.66% |
| P16 | PBL | chr3 | 10188224 | 10188224 | G | - | NM_000551 | VHL | frameshift_variant | p.T124Hfs*35 | c.369del | 11 | 6630 | 0.17% |
| P16 | PBL | chr3 | 52441217 | 52441217 | C | T | NM_004656 | BAP1 | missense_variant | p.G185R | c.553G>A | 0 | 4679 | 0.00% |
| P16 | Plasma1 | chr3 | 52441217 | 52441217 | C | T | NM_004656 | BAP1 | missense_variant | p.G185R | c.553G>A | 248 | 4519 | 5.49% |
| P16 | Tissue | chr3 | 52441217 | 52441217 | C | T | NM_004656 | BAP1 | missense_variant | p.G185R | c.553G>A | 49 | 95 | 51.58% |
| P16 | Plasma2 | chr3 | 52441217 | 52441217 | C | T | NM_004656 | BAP1 | missense_variant | p.G185R | c.553G>A | 0 | 940 | 0.00% |
| P16 | PBL | chr3 | 52696229 | 52696229 | C | A | NM_181042 | PBRM1 | stop_gained | p.E150* | c.448G>T | 3 | 4935 | 0.06% |
| P16 | Plasma1 | chr3 | 52696229 | 52696229 | C | A | NM_181042 | PBRM1 | stop_gained | p.E150* | c.448G>T | 458 | 6596 | 6.94% |
| P16 | Tissue | chr3 | 52696229 | 52696229 | C | A | NM_181042 | PBRM1 | stop_gained | p.E150* | c.448G>T | 46 | 85 | 54.12% |
| P16 | Plasma2 | chr3 | 52696229 | 52696229 | C | A | NM_181042 | PBRM1 | stop_gained | p.E150* | c.448G>T | 9 | 1449 | 0.62% |
| P16 | Tissue | chr5 | 180432665 | 1.8E+08 | C | - | NM_197975 | BTNL3 | frameshift_variant | p.S401Afs*7 | c.1200del | 9 | 210 | 4.29% |
| P16 | Plasma1 | chr5 | 180432665 | 1.8E+08 | C | - | NM_197975 | BTNL3 | frameshift_variant | p.S401Afs*7 | c.1200del | 223 | 15223 | 1.46% |
| P16 | Plasma2 | chr5 | 180432665 | 1.8E+08 | C | - | NM_197975 | BTNL3 | frameshift_variant | p.S401Afs*7 | c.1200del | 37 | 3056 | 1.21% |
| P16 | PBL | chr5 | 180432665 | 1.8E+08 | C | - | NM_197975 | BTNL3 | frameshift_variant | p.S401Afs*7 | c.1200del | 65 | 9080 | 0.72% |
| P18 | PBL | chr3 | 10183809 | 10183809 | G | A | NM_000551 | VHL | missense_variant | p.G93D | c.278G>A | 1 | 4799 | 0.02% |
| P18 | Plasma1 | chr3 | 10183809 | 10183809 | G | A | NM_000551 | VHL | missense_variant | p.G93D | c.278G>A | 0 | 2512 | 0.00% |
| P18 | Tissue | chr3 | 10183809 | 10183809 | G | A | NM_000551 | VHL | missense_variant | p.G93D | c.278G>A | 801 | 2000 | 40.05% |
| P18 | Tissue | chr3 | 52588796 | 52588796 | G | - | NM_018313 | PBRM1 | frameshift_variant | p.P1411Lfs*21 | c.4232del | 276 | 4481 | 6.16% |
| P18 | Plasma1 | chr3 | 52588796 | 52588796 | G | - | NM_018313 | PBRM1 | frameshift_variant | p.P1411Lfs*21 | c.4232del | 7 | 9363 | 0.07% |
| P18 | PBL | chr3 | 52588796 | 52588796 | G | - | NM_018313 | PBRM1 | frameshift_variant | p.P1411Lfs*21 | c.4232del | 17 | 8276 | 0.21% |
| P18 | PBL | chr3 | 52588847 | 52588847 | A | C | NM_018313 | PBRM1 | missense_variant | p.V1394G | c.4181T>G | 0 | 5793 | 0.00% |
| P18 | Plasma1 | chr3 | 52588847 | 52588847 | A | C | NM_018313 | PBRM1 | missense_variant | p.V1394G | c.4181T>G | 0 | 5446 | 0.00% |
| P18 | Tissue | chr3 | 52588847 | 52588847 | A | C | NM_018313 | PBRM1 | missense_variant | p.V1394G | c.4181T>G | 203 | 3130 | 6.49% |
| P18 | Tissue | chr3 | 52610623 | 52610623 | - | T | NM_181042 | PBRM1 | frameshift_variant | p.M1209Nfs*11 | c.3625dup | 669 | 4445 | 15.05% |
| P18 | Plasma1 | chr3 | 52610623 | 52610623 | - | T | NM_181042 | PBRM1 | frameshift_variant | p.M1209Nfs*11 | c.3625dup | 8 | 7356 | 0.11% |
| P18 | PBL | chr3 | 52610623 | 52610623 | - | T | NM_181042 | PBRM1 | frameshift_variant | p.M1209Nfs*11 | c.3625dup | 10 | 8516 | 0.12% |
| P18 | Tissue | chr3 | 52613207 | 52613210 | TTCT | - | NM_181042 | PBRM1 | frameshift_variant | p.E1132Mfs*26 | c.3393_3396del | 327 | 3797 | 8.61% |
| P18 | Plasma1 | chr3 | 52613207 | 52613210 | TTCT | - | NM_181042 | PBRM1 | frameshift_variant | p.E1132Mfs*26 | c.3393_3396del | 1 | 6114 | 0.02% |
| P18 | PBL | chr3 | 52613207 | 52613210 | TTCT | - | NM_181042 | PBRM1 | frameshift_variant | p.E1132Mfs*26 | c.3393_3396del | 8 | 6808 | 0.12% |
| P18 | Tissue | chr3 | 52668809 | 52668809 | C | - | NM_181042 | PBRM1 | frameshift_variant | p.E370Dfs*34 | c.1110del | 143 | 4420 | 3.24% |
| P18 | Plasma1 | chr3 | 52668809 | 52668809 | C | - | NM_181042 | PBRM1 | frameshift_variant | p.E370Dfs*34 | c.1110del | 0 | 5874 | 0.00% |
| P18 | PBL | chr3 | 52668809 | 52668809 | C | - | NM_181042 | PBRM1 | frameshift_variant | p.E370Dfs*34 | c.1110del | 6 | 8202 | 0.07% |
| P18 | Tissue | chr3 | 52713696 | 52713696 | G | - | NM_181042 | PBRM1 | frameshift_variant | p.P11Lfs*34 | c.32del | 261 | 4077 | 6.40% |
| P18 | Plasma1 | chr3 | 52713696 | 52713696 | G | - | NM_181042 | PBRM1 | frameshift_variant | p.P11Lfs*34 | c.32del | 6 | 8249 | 0.07% |
| P18 | PBL | chr3 | 52713696 | 52713696 | G | - | NM_181042 | PBRM1 | frameshift_variant | p.P11Lfs*34 | c.32del | 8 | 7804 | 0.10% |
| P18 | Tissue | chrX | 53227012 | 53227012 | C | - | NM_004187 | KDM5C | frameshift_variant | p.A855Pfs*6 | c.2563del | 93 | 2762 | 3.37% |
| P18 | Plasma1 | chrX | 53227012 | 53227012 | C | - | NM_004187 | KDM5C | frameshift_variant | p.A855Pfs*6 | c.2563del | 1 | 4220 | 0.02% |
| P18 | PBL | chrX | 53227012 | 53227012 | C | - | NM_004187 | KDM5C | frameshift_variant | p.A855Pfs*6 | c.2563del | 7 | 3507 | 0.20% |
| P18 | PBL | chrX | 53230855 | 53230855 | C | G | NM_004187 | KDM5C | missense_variant | p.E646D | c.1938G>C | 0 | 2355 | 0.00% |
| P18 | Plasma1 | chrX | 53230855 | 53230855 | C | G | NM_004187 | KDM5C | missense_variant | p.E646D | c.1938G>C | 0 | 1995 | 0.00% |
| P18 | Tissue | chrX | 53230855 | 53230855 | C | G | NM_004187 | KDM5C | missense_variant | p.E646D | c.1938G>C | 41 | 1889 | 2.17% |
| P18 | PBL | chrX | 53241010 | 53241010 | C | T | NM_004187 | KDM5C | missense_variant | p.A401T | c.1201G>A | 1 | 2841 | 0.04% |
| P18 | Plasma1 | chrX | 53241010 | 53241010 | C | T | NM_004187 | KDM5C | missense_variant | p.A401T | c.1201G>A | 0 | 2711 | 0.00% |
| P18 | Tissue | chrX | 53241010 | 53241010 | C | T | NM_004187 | KDM5C | missense_variant | p.A401T | c.1201G>A | 137 | 2050 | 6.68% |
| P18 | PBL | chrX | 53247098 | 53247098 | C | T | NM_004187 | KDM5C | stop_gained | p.W134* | c.402G>A | 0 | 2251 | 0.00% |
| P18 | Plasma1 | chrX | 53247098 | 53247098 | C | T | NM_004187 | KDM5C | stop_gained | p.W134* | c.402G>A | 0 | 1803 | 0.00% |
| P18 | Tissue | chrX | 53247098 | 53247098 | C | T | NM_004187 | KDM5C | stop_gained | p.W134* | c.402G>A | 164 | 1708 | 9.60% |
| P18 | Tissue | chrX | 53250031 | 53250044 | TTCAGCCTCTGGAT | - | NM_004187 | KDM5C | frameshift_variant | p.I69* | c.205_218del | 92 | 2638 | 3.49% |
| P18 | Plasma1 | chrX | 53250031 | 53250044 | TTCAGCCTCTGGAT | - | NM_004187 | KDM5C | frameshift_variant | p.I69* | c.205_218del | 2 | 4480 | 0.04% |
| P18 | PBL | chrX | 53250031 | 53250044 | TTCAGCCTCTGGAT | - | NM_004187 | KDM5C | frameshift_variant | p.I69* | c.205_218del | 0 | 3072 | 0.00% |
| B-1 | T3 | chr17 | 7579410 | 7579411 | G | - | NM_000546 | TP53 | frameshift_variant | p.L93Cfs*30 | c.277del | 2 | 3916 | 0.05% |
| B-1 | T3 | chr17 | 7579410 | 7579411 | G | - | NM_000546 | TP53 | frameshift_variant | p.L93Cfs*30 | c.277del | 37 | 3569 | 1.04% |
| B-1 | T3 | chr17 | 7579410 | 7579411 | G | - | NM_000546 | TP53 | frameshift_variant | p.L93Cfs*30 | c.277del | 321 | 5425 | 5.92% |
| B-1 | T3 | chr3 | 178936082 | 1.79E+08 | G | A | NM_006218 | PIK3CA | missense_variant | p.E542K | c.1624G>A | 0 | 7413 | 0.00% |
| B-1 | T3 | chr3 | 178936082 | 1.79E+08 | G | A | NM_006218 | PIK3CA | missense_variant | p.E542K | c.1624G>A | 164 | 6827 | 2.40% |
| B-1 | T3 | chr3 | 178936082 | 1.79E+08 | G | A | NM_006218 | PIK3CA | missense_variant | p.E542K | c.1624G>A | 443 | 7294 | 6.07% |
| B-1 | T4 | chr3 | 10188253 | 10188257 | AACT | - | NM_000551 | VHL | frameshift_variant | p.T133Nfs*25 | c.396_399del | 0 | 2394 | 0.00% |
| B-1 | T4 | chr3 | 10188253 | 10188257 | AACT | - | NM_000551 | VHL | frameshift_variant | p.T133Nfs*25 | c.396_399del | 330 | 4504 | 7.33% |
| B-1 | T4 | chr3 | 10188253 | 10188257 | AACT | - | NM_000551 | VHL | frameshift_variant | p.T133Nfs*25 | c.396_399del | 666 | 5050 | 13.19% |
| A-1 | T3 | chr17 | 7577539 | 7577539 | G | A | NM_000546 | TP53 | missense_variant | p.R248W | c.742C>T | 0 | 3232 | 0.00% |
| A-1 | T3 | chr17 | 7577539 | 7577539 | G | A | NM_000546 | TP53 | missense_variant | p.R248W | c.742C>T | 165 | 5484 | 3.01% |
| A-1 | T3 | chr17 | 7577539 | 7577539 | G | A | NM_000546 | TP53 | missense_variant | p.R248W | c.742C>T | 0 | 4796 | 0.00% |
| A-2 | T3 | chr1 | 11188177 | 11188177 | T | A | NM_004958 | MTOR | missense_variant | p.I1973F | c.5917A>T | 4 | 6271 | 0.06% |
| A-2 | T3 | chr1 | 11188177 | 11188177 | T | A | NM_004958 | MTOR | missense_variant | p.I1973F | c.5917A>T | 397 | 6252 | 6.35% |
| A-2 | T3 | chr1 | 11188177 | 11188177 | T | A | NM_004958 | MTOR | missense_variant | p.I1973F | c.5917A>T | 0 | 5831 | 0.00% |
| A-2 | T4 | chr1 | 27094317 | 27094317 | - | C | NM_006015 | ARID1A | frameshift_variant | p.K1010Qfs*4 | c.3027dup | 0 | 3537 | 0.00% |
| A-2 | T4 | chr1 | 27094317 | 27094317 | - | C | NM_006015 | ARID1A | frameshift_variant | p.K1010Qfs*4 | c.3027dup | 310 | 7054 | 4.39% |
| A-2 | T4 | chr1 | 27094317 | 27094317 | - | C | NM_006015 | ARID1A | frameshift_variant | p.K1010Qfs*4 | c.3027dup | 0 | 2800 | 0.00% |
| A-2 | T4 | chr1 | 27105559 | 27105559 | C | A | NM_006015 | ARID1A | missense_variant | p.L1724M | c.5170C>A | 0 | 3698 | 0.00% |
| A-2 | T4 | chr1 | 27105559 | 27105559 | C | A | NM_006015 | ARID1A | missense_variant | p.L1724M | c.5170C>A | 39 | 4039 | 0.97% |
| A-2 | T4 | chr1 | 27105559 | 27105559 | C | A | NM_006015 | ARID1A | missense_variant | p.L1724M | c.5170C>A | 0 | 3323 | 0.00% |
